# Benchmark datasets for 3D MALDI- and DESI-imaging mass spectrometry

**DOI:** 10.1186/s13742-015-0059-4

**Published:** 2015-05-04

**Authors:** Janina Oetjen, Kirill Veselkov, Jeramie Watrous, James S McKenzie, Michael Becker, Lena Hauberg-Lotte, Jan Hendrik Kobarg, Nicole Strittmatter, Anna K Mróz, Franziska Hoffmann, Dennis Trede, Andrew Palmer, Stefan Schiffler, Klaus Steinhorst, Michaela Aichler, Robert Goldin, Orlando Guntinas-Lichius, Ferdinand von Eggeling, Herbert Thiele, Kathrin Maedler, Axel Walch, Peter Maass, Pieter C Dorrestein, Zoltan Takats, Theodore Alexandrov

**Affiliations:** 1 MALDI Imaging Lab, University of Bremen, Bremen, Germany; 2 Department of Surgery and Cancer, Faculty of Medicine, Imperial College London, London, UK; 3 Department of Medicine, Biomedical Research Facility II, University of California, San Diego, USA; 4 Bruker Daltonik GmbH, Bremen, Germany; 5 Steinbeis Center SCiLS Research, Bremen, Germany; 6 Institute of Physical Chemistry, Friedrich-Schiller-University Jena, Jena, Germany; 7 Department of Otorhinolaryngology, Jena University Hospital, Jena, Germany; 8 SCiLS GmbH, Bremen, Germany; 9 European Molecular Biology Laboratory, Heidelberg, Germany; 10 Research Unit Analytical Pathology, Institute of Pathology, Helmholtz Center Munich, Munich, Germany; 11 Department of Medicine, Faculty of Medicine, Imperial College London, London, UK; 12 Leibnitz Institute of Photonic Technology (IPHT), Jena, Germany; 13 Jena Center for Soft Matter (JCSM), Friedrich-Schiller-University Jena, Jena, Germany; 14 Islet Research Lab, Center for Biomolecular Interactions, University of Bremen, Bremen, Germany; 15 Center for Industrial Mathematics, University of Bremen, Bremen, Germany; 16 Skaggs School of Pharmacy & Pharmaceutical Sciences, University of California, San Diego, USA

**Keywords:** Benchmark datasets, 3D imaging mass spectrometry, Three-dimensional, MALDI, DESI, imzML

## Abstract

**Background:**

Three-dimensional (3D) imaging mass spectrometry (MS) is an analytical chemistry technique for the 3D molecular analysis of a tissue specimen, entire organ, or microbial colonies on an agar plate. 3D-imaging MS has unique advantages over existing 3D imaging techniques, offers novel perspectives for understanding the spatial organization of biological processes, and has growing potential to be introduced into routine use in both biology and medicine. Owing to the sheer quantity of data generated, the visualization, analysis, and interpretation of 3D imaging MS data remain a significant challenge. Bioinformatics research in this field is hampered by the lack of publicly available benchmark datasets needed to evaluate and compare algorithms.

**Findings:**

High-quality 3D imaging MS datasets from different biological systems at several labs were acquired, supplied with overview images and scripts demonstrating how to read them, and deposited into MetaboLights, an open repository for metabolomics data. 3D imaging MS data were collected from five samples using two types of 3D imaging MS. 3D matrix-assisted laser desorption/ionization imaging (MALDI) MS data were collected from murine pancreas, murine kidney, human oral squamous cell carcinoma, and interacting microbial colonies cultured in Petri dishes. 3D desorption electrospray ionization (DESI) imaging MS data were collected from a human colorectal adenocarcinoma.

**Conclusions:**

With the aim to stimulate computational research in the field of computational 3D imaging MS, selected high-quality 3D imaging MS datasets are provided that could be used by algorithm developers as benchmark datasets.

**Electronic supplementary material:**

The online version of this article (doi:10.1186/s13742-015-0059-4) contains supplementary material, which is available to authorized users.

## Data description

Three-dimensional imaging mass spectrometry (3D imaging MS) is a spatially resolved analytical technique for three-dimensional molecular analysis of a tissue specimen, entire organ, or agar plate. 3D imaging MS can image the spatial distribution of thousands of molecules such as proteins, peptides, lipids, and small molecules [1]. Usually, 3D imaging MS is performed by serial sectioning of a sample followed by two-dimensional (2D) imaging MS analysis of each section. 2D imaging MS is an established technique of analytical chemistry for surface molecular analysis with various applications in biology and medicine [2]. 2D imaging MS collects mass spectra pixel by pixel over the sample surface. For each pixel, the mass spectrum represents the intensities of thousands to millions of mass-to-charge (*m/z*) values, which depends on the sampling rate of the detector and the mass resolving power of the instrument. The intensity at an *m*/*z*-value is proportional to the number of ions with this *m*/*z*-value that are desorbed from the area of the sample surface corresponding to the respective pixel.

Various ionization sources and mass spectrometric techniques have been coupled and developed for imaging MS and, consequently, for serial sectioning-based 3D imaging MS; see [3,4] for a review. Two different ionization techniques have been used to acquire the data provided by us: matrix-assisted laser desorption/ionization (MALDI) and desorption electro spray ionization (DESI). In MALDI imaging MS, a small organic compound, the so-called matrix, is applied to the surface of a section, usually in a solution with an organic solvent. The matrix has two functions: first, the organic solvent helps to extract analytes from the sample, which then cocrystallize with the matrix compound; second, the matrix helps to softly dissipate the energy from high-frequency laser pulses to the sample to desorb and ionize intact analytes from the sample surface [5-7].

DESI-imaging MS uses another principle for producing ions and runs under atmospheric pressure [8]. A pneumatically assisted electrospray is directed onto the sample surface where it generates a liquid film that desorbs analytes from the sample surface. Upon impact of further primary droplets, secondary droplets containing analyte molecules are ejected from the liquid film and subsequently sampled by an extended mass spectrometer inlet capillary (a so-called sniffer).

In both ionization techniques, ions are formed from a small area of the sample surface, and these are directed into the mass spectrometer. A movable stage translates the sample under the ionization probe to acquire mass spectra from the different raster positions (pixels) across the sample.

An imaging MS dataset can be considered as a datacube or hyperspectral image with spectra assigned with spatial *x*- and *y*-coordinates, or molecular ion images, each representing relative intensities of ions with a specific *m*/*z* value [9]. Imaging MS enables one either to visualize the spatial distribution of a particular ion within the section or to evaluate the molecular composition at a particular pixel. Analysis and interpretation of high-dimensional imaging MS data require automated computational methods [10-13], and 3D imaging MS leads to additional computational challenges as one dataset encompasses 10–100 imaging MS datasets of serial sections.

In this data note, a total of five 3D imaging MS datasets in the imzML format (an open and standard file format for imaging MS data [14]) are provided and available for download in the MetaboLights repository [MTBLS176], as well as the *GigaScience* GigaDB respository [15]. The imzML file structure consists of an XML-like file containing metadata (*.imzML) and a binary data file containing spectra (*.ibd); both are unequivocally linked by a universally unique identifier. In the imzML files provided here, the relative position of each voxel in the 3D space is stored in the “userParam” field.

The 3D DESI-imaging MS dataset is provided both in multiple imzML files each containing an 2D imaging MS dataset of an individual section and in a single HDF5 [16] file containing the metadata, coregistered imaging MS data, and optical [haematoxylin and eosin (H&E)-stained] images.

The data-acquisition parameters are briefly described in the following section. General information about each dataset can be found in Additional file 1. An overview showing intensity distributions for exemplary *m*/*z*-values together with the mean spectrum for each dataset is provided in Additional file 2.

### 3D MALDI imaging MS dataset of a mouse kidney

The dataset comprises 75 sections from the central part of a mouse kidney that was PAXgene® fixed and paraffin embedded. As such, it is a part of the kidney dataset that was presented in a previous publication to demonstrate the experimental and computational pipeline for 3D imaging MS [17]. However, the dataset itself was never published. Microtome sections with a thickness of 3.5 μm were covered with 10 mg/ml of sinapinic acid (SA) in 60% acetonitrile and 0.2% trifluoroacetic acid as matrix after paraffin removal and washing as described previously [17]. The matrix was applied using a vaporization sprayer (ImagePrep™, Bruker Daltonics, Bremen, Germany). Spectra were acquired using a Bruker Daltonics Autoflex speed™ MALDI mass spectrometer in linear positive mode in the mass range of 2,000-20,000 *m/z* and a deflection of 1,500 *m/z*. In total, the dataset comprised 1,362,830 spectra, each containing 7,680 data points. Each spectrum was acquired with 200 laser shots, and the random walk option was set to 20 shots per position. A medium-size laser focus was chosen, so as to be suitable for the selected lateral resolution of 50 μm pixel size. During the data acquisition, the spectra preprocessing included a Gaussian spectral smoothing with a width of 2 within 4 cycles as well as baseline reduction using the Top Hat algorithm. The data for all 75 sections were imported into the software SCiLS Lab (SCiLS, Bremen, Germany) version 2014b. The registration of individual sections was performed with the aim to reconstruct the original relations between the sections. For this purpose, the so-called user-guided rigid registration was used, and this was performed interactively as follows. First, the first of the consecutive sections was placed in the center of the software view. Then, each of the following sections was positioned over the previous image and moved in the *x*- and *y*-directions and rotated with the help of the interactive software (keyboard, mouse); the half-transparent overlap with the previous image helps evaluate the positioning. The method allows for compensation of rotations and translations. Finally, the dataset containing spectra with adjusted spatial coordinates *x* and *y* and newly assigned coordinate *z* was exported into the imzML format with files named 3DMouseKidney.ibd and 3DMouseKidney.imzML. These files are described in the corresponding Readme (Additional file 3). A visualization of the 3D mouse kidney dataset performed in the software SCiLS Lab, version 2014b is shown in Additional file 2: Figure S1.

### 3D MALDI imaging MS dataset of a mouse pancreas

The 3D mouse pancreas dataset was created in a similar fashion to the mouse kidney dataset. A C57BL/6 mouse was sacrificed, and the pancreas was immediately isolated, fixed in PAXgene® Tissue Containers according to the manufacturer’s instructions (Qiagen, Hilden, Germany), dehydrated, and embedded in low-melting-point paraffin as described previously [17]. Sections (5 μm in thickness) were cut on a microtome and mounted on indium-tin-coated conductive glass slides (Bruker Daltonics). After paraffin removal and washing, 2,5-dihydroxybenzoic acid (DHB), dissolved at 30 mg/ml in 50% methanol with 0.2% TFA as a matrix, was used. Spectra from 29 consecutive sections were acquired using a Bruker Daltonics Autoflex speed™ mass spectrometer in linear positive mode in the mass range 1,600-15,000 *m/z*. A medium-size laser diameter was used, with a lateral resolution of 60 μm and 500 laser shots per pixel were accumulated with the random walk option set to 100 shots per position. The complete dataset with 29 sections comprised 497,225 spectra with 13,312 data points per spectrum. The unprocessed raw data were imported into the software SCiLS Lab, version 2014b. For 3D image registration in SCiLS Lab, a section thickness of 5 μm was selected. The image registration was performed as described earlier for the 3D mouse kidney. Data conversion into the imzML format was performed as described for the mouse kidney above, and the files which are described in Additional file 4 were named 3D_Mouse_Pancreas.ibd and 3D_Mouse_Pancreas.imzML. A visualization of the 3D mouse pancreas dataset is shown in Additional file 2: Figure S2.

### 3D MALDI imaging MS dataset of a human oral squamous cell carcinoma

A tissue specimen from a patient with an oral squamous cell carcinoma (OSCC) was obtained from the Department of Otorhinolaryngology, University Hospital Jena. The necessary approval was obtained from the local Ethics Committee, approval No. 3008-12/10.

3D MALDI imaging MS analysis was applied to 58 cryosections, each with a thickness of 10 μm. The sections were mounted on indium-tin-oxide-coated conductive glass slides (Bruker Daltonics) and stored at −80°C until use. After drying under vacuum for 15 min, the slides were washed twice for 2 min in 70% ethanol and thereafter for 2 min in 99% ethanol. The SA used as a matrix was applied using the Bruker ImagePrep™ device. MALDI imaging MS was performed on an Autoflex speed™ mass spectrometer (Bruker Daltonics) in linear positive mode. Spectra were acquired in the mass range 2,000-20,000 *m/z* with a deflection set to 1,500 *m/z*. Each spectrum was a sum of 200 laser shots, and the random walk option was set to 25 shots per position. A medium-size laser diameter was selected for the chosen lateral resolution of 60 μm. In total, the dataset comprised 828,558 spectra with 7,680 data points per spectrum. The spectra were preprocessed during acquisition applying Gaussian spectral smoothing with a width of 2 within 4 cycles as well as baseline reduction using the Top Hat algorithm. The data for all sections were imported into the software SCiLS Lab, version 2014b, and rigid image registration was performed by user-guided stacking of the optical images as described earlier for the 3D mouse kidney dataset. A slice thickness, or *z*-distance, of 60 μm was selected to produce voxels of 60 μm^3^. Finally, the dataset was exported to the imzML format producing files 3D_OSCC.ibd and 3D_OSCC.imzML as described in Additional file 5. A visualization of the 3D human OSCC dataset is shown in Additional file 2: Figure S3.

### 3D MALDI imaging MS datasets of cultured microbial colonies in a time course experiment

3D MALDI imaging MS is very suitable for studying the metabolic exchange between interacting microbes [18,19]. For this dataset, metabolic exchange of the interacting microbes *Streptomyces coelicolor* A3(2) and *Bacillus subtilis* PY79 was followed in a time-course experiment on the first, fourth, and eighth days after co-inoculation in a Petri dish. Culturing of the microbes and sample preparation for 3D MALDI imaging MS were performed as described elsewhere [19]. Briefly, equally sized agar slices were sectioned and mounted on a MALDI-TOF steel target. A universal matrix (a mixture of alpha-cyano-4-hydroxycinnamic acid and 2,5-dihydroxybenzoic acid) was applied with a 50 μm pore size sieve, and the samples were allowed to dry completely. Spectra were acquired on an Autoflex™ MALDI-TOF mass spectrometer (Bruker) in linear positive mode in the mass range of 0–4,000 *m/z* using a large laser diameter and 300 shots per spectrum. A lateral resolution of 400 μm was selected. All individual sections were imported into the software SCiLS Lab, version 2014b, for 3D volume generation. In total, the dataset comprised 17,672 spectra, and the bin size was reduced to 40,299 data points per spectrum during import. To construct a 3D volume that resembled the length, width, and height of the original agar block, a thickness of 1,500 μm per section producing voxels of 400 × 400 × 1,500 μm was chosen. The 3D volume was built up, starting with the first section from the day 1 post-inoculation dataset. After completion of image registration from the first time point, a spacing of 10.5 mm was introduced, starting with the block from the time point day 4. The same steps were repeated for the block from time point day 8 after inoculation. Besides these additional steps, the image registration was performed as described earlier for the 3D mouse kidney dataset. The complete dataset was then exported into the imzML format to produce the files Microbe_Interaction_3D_Timecourse_LP.ibd and Microbe_Interaction_3D_Timecourse_LP.imzML which are described in the corresponding Readme file (Additional file 6). A visualization of the 3D dataset of the microbial colonies in a time-course experiment is shown in Additional file 2: Figure S4.

### 3D DESI-imaging MS dataset of a human colorectal adenocarcinoma

Sections from a single colorectal adenocarcinoma (n = 26) were analyzed by DESI-imaging MS. The tissue specimen was snap-frozen in liquid nitrogen and stored in a freezer at −80°C prior to cryosectioning at 10 μm thickness using a Microm HM550 Cryostat (Thermo Fisher Scientific, Runcorn, UK) set at −16°C, and thaw mounted onto SuperFrost® Glass slides (Thermo Fisher Scientific). Distilled water was used to mount the sample to the sample holder, and the cryosectioning was performed without embedding medium. The built-in vacutome function of the cryostat was used to facilitate sectioning. The slides were stored in closed containers at −80°C prior to analysis and allowed to thaw at room temperature under nitrogen flow prior to DESI-imaging MS acquisition.

Sections were cut to a step size of 10 μm, and every tenth section was imaged. Four sequential sections were deposited on each slide. The instrumental spatial resolution was set to 100 μm, and analysis of every tenth 10 μm section resulted in 100 μm^3^ voxels.

Imaging MS data were acquired in the negative-ion mode over an *m/z* range of 200–1,050 using a Thermo Exactive instrument (Thermo Scientific GmbH, Bremen, Germany) coupled to a home-built automated DESI-imaging source as described previously [20]. The solvent used for DESI analysis was methanol/water (95/5 v/v) at a flow rate of 1.5 ml/min. Nitrogen was used as a nebulizing gas at a pressure of 7 bar. The distance between the DESI spray tip and the sample surface was set to 1.5 mm; the distance between the DESI spray tip and the mass spectrometer was set to 14 mm; and the distance between the inlet capillary and the sample surface was 0.1 mm. The spray angle was 80°, whereas the collection angle was fixed at 10°. The spray voltage used for analysis was 4.5 kV. Each row of pixels was acquired as a continuous line scan over the sample surface and saved in a separate raw file. All Thermo raw files of one imaging experiment were then converted to imzML format using the imzML converter v1.1.4.5i [21]. The imzML files were named with reference to the section number and location of the section on the slide. For example, in the file named “120TopL, 90TopR, 110BottomL, 100BottomR-centroid.imzML”, the top-right section was the 90th section cut from the sample at a depth of 900 μm. A more detailed description can be found in Additional file 7.

Following imaging, the sections were stained with H&E. A consultant histopathologist assessed the samples for histological tissue types (independently of the results of DESI-imaging). The sample was found to consist mainly of two tissue types: tumor and connective tissue. H&E scanned sections were digitalized using a Nanozoomer 2.0-HT C9600 slide scanning instrument (Hamamatsu Photonics, Hamamatsu City, Japan).

In addition to providing imzML files, each storing imaging MS data of an individual serial section, the full dataset was provided after several processing steps (see below) in an HDF5 file. A description of the HDF5 file can be found in Additional file 8. HDF5 is a flexible and platform independent format for storing large datasets; for more information on HDF5, see [16] along with example code for a range of programming languages. The GitHub repository (see [22]) contains a MATLAB function (import3dh5.m) that can be used to import the data and provide some context to the MATLAB functions used for reading HDF5 files (for example, h5readatt, h5read, h5info). Data within the HDF5 file are arranged as follows: the *m/z* vector is stored at “/mz” and data from the nth slice can be found in the “/data/sn” group. Each of these groups contains the optical image (“/data/sn/op”), MS image (“/data/sn/x”) and the section number (“/data/sn/zPosition”). Sample metadata are stored in the root directory (“/”).

The compilation of 3D DESI-imaging MS dataset into the HDF5 file included the following preprocessing stages: (a) matching of peak lists within and between all tissue sections; (b) separation of neighboring tissue sections into separate imaging MS datasets; (c) automated co-registration of histological and MS images for 3D dataset compilation; and (d) spectral normalization to account for overall intensity bias between spectral profiles. The resulting workflow for 3D DESI-imaging MS dataset compilation was devised based on image alignment and peak matching algorithms published previously [23].

- Owing to inherent variability in mass detection, molecular ion species within an *m/z* range smaller than the native accuracy of the mass spectrometer (<5 ppm in our case) were assigned to the same molecular ion species uniformly for all pixels across tissue sections.
- In order to be able to divide the slides properly into separate sections, the optical and MS images were aligned by means of overlap between tissue object pixels in MS and optical images. The aligned optical image was thus a warped form of the original (the MS image remains static) by means of affine transformation as previously described [23]. Four polygons were drawn over the newly aligned optical image, and these regions were exported to individual files.
- The individual MS imaging datasets were aligned to each other. By default, the procedure was started with the first slice (that is, slice number 10), which was used as the template image and was the only image that remained unchanged. The procedure was for the optical image of the subsequent section to be co-registered with the optical image of the preceding slice (fixed), and the required transformation was applied to both MS and optical images. These newly transformed images thus formed the template for the subsequent slice. The process was continued until the last slice was reached. As a consequence of the alignment, all of the optical images had the same dimensions, as did the MS images. For more information on the co-registration and transformation used for this dataset, please refer to [23].

Median fold change normalization was finally applied to reduce any variation in overall signal intensity between spectral profiles within and between tissue samples. An illustration of the 3D DESI-imaging MS dataset of a colorectal adenocarcinoma visualizing the distributions of two exemplary *m*/*z*-values is shown in Additional file 2: Figure S5.

### Instructions for loading the imzML files

Currently, there is no 3D-oriented data format for storing 3D imaging MS data and no free software for loading and visualizing 3D imaging MS data. Data were provided in the imzML format, an open and community-accepted format for exchange of imaging MS data, and for each spectrum the user-defined parameters of its location in 3D space were introduced. For more information on the imzML format, including instructions on how to read it, please refer to [21]. Several freely available software packages are available for reading 2D imzML files, including BioMap [24], Datacube Explorer [25], and MSiReader [26]. However, these software packages do not allow one to open datasets that are as large as those provided here and are for 2D data only. The Volume Explorer software was developed at FOM Institute AMOLF for 3D imaging MS data analysis and visualization; it is not available for download but was reported to be available on request [25].

The datasets are available for download in the MetaboLights repository [MTBLS176], as well as the *GigaScience* GigaDB respository [15]. For loading data from the provided datasets, a script that can load individual spectra or images is provided. The script uses a Java based imzML data parser freely available at [27] as a part of the imzMLConverter Java package [28]. The script for each MALDI imaging MS dataset (3D kidney, 3D pancreas, 3D OSCC, 3D time course) was adapted, and this was provided as Additional files 9, 10, 11, and 12.

### Data quality

For 3D imaging MS, the reproducibility of the measurements for the individual section is of high importance. Currently, there are no quality-control standards either for 2D or for 3D imaging MS data. In our experiments, the quality control began with a visual evaluation of the integrity of each serial section. Where applicable, controlled conditions for matrix application for the MALDI imaging MS datasets were used to guarantee equal amounts of matrix and a homogenous matrix layer, a prerequisite for reproducible spectra quality. The instrument acquisition parameters and experimental conditions for DESI-imaging MS were kept consistent across all adjacent tissue sections to minimize any unwanted variation. The spectra quality was ascertained by manual acquisition of test spectra from each section before starting the automatic acquisition, and calibration standards were used to reduce sectionwide peak shifts. Selected spectra and images from all datasets were visually inspected, and it was checked whether known anatomical structures were detectable based on *m/z* values or cluster map analysis.

### Potential use

The main aim of this data note is to stimulate bioinformatic developments in the new, promising, and challenging field of 3D imaging MS by providing the bioinformatics community with several high-quality 3D imaging MS datasets representing different samples and types of mass spectrometry. We encourage bioinformaticians to develop algorithms for efficient spectral processing specifically for 3D imaging MS.

Analyzing 3D imaging MS data is challenging because of the complexity, 3D-dimensionality and size. The size of a 3D imaging MS dataset can be as high as 100 GB, depending on the instrument’s resolving power. The size will only increase with the introduction into 3D imaging MS of ultrahigh-resolution mass spectrometry, such as Fourier transform-ion cyclotron resonance or Orbitrap. This large dataset requires efficient algorithms potentially integrated with data-compression methods to aid data storage and to facilitate data querying, analysis, and visualization, to be performed in the cloud, on a server, or on a personal workstation.

Note that 3D imaging MS data are prone to considerable variability, because the sectionwide analysis and long acquisition time span several days, or sometimes weeks. The development of methods compensating for these effects would increase the reproducibility of the experiments. This includes normalization, baseline correction, noise reduction, and, in particular, peak alignment that needs to be performed on a large number of spectra with the peaks between sections expected to be misaligned to a higher degree than within one section.

As for 2D imaging MS data analysis, there is still a need for open-access software tools for the analysis of 3D imaging MS data, including dimensionally reduction algorithms and methods for unsupervised and supervised data analysis.

By making our datasets available for the community, we aim to stimulate the development, evaluation, and comparison of novel and efficient algorithms for analysis and interpretation of large 3D imaging MS datasets.

Another aim for sharing the datasets is to facilitate inter-laboratory comparisons of 3D imaging MS datasets, essential for raising the level of the technology and paving the way to open-access science.

## Availability of supporting data

The datasets supporting the results of this article are available in the MetaboLights repository [MTBLS176], as well as the *GigaScience* GigaDB respository [15].

## Additional files

Additional file 1:
**Information about the 3D imaging MS datasets.**
Additional file 2:
**Supplementary information with figures S1-S5.**
Additional file 3:
**Readme file for the 3D MALDI imaging MS dataset of a mouse kidney.**
Additional file 4:
**Readme file for the 3D MALDI imaging MS dataset of a mouse pancreas.**
Additional file 5:
**Readme file for the 3D MALDI imaging MS dataset of a human oral squamous cell carcinoma.**
Additional file 6:
**Readme file for the 3D MALDI imaging MS dataset of microbial colonies in a time course experiment.**
Additional file 7:
**Readme file for the individual 2D DESI imaging MS datasets of a human colorectal adenocarcinoma.**
Additional file 8:
**Readme file for the 3D DESI imaging MS dataset of a human colorectal adenocarcinoma.**
Additional file 9:
**Script for the 3D MALDI imaging MS dataset of a mouse kidney.**
Additional file 10:
**Script for the 3D MALDI imaging MS dataset of a mouse pancreas.**
Additional file 11:
**Script for the 3D MALDI imaging MS dataset of a human oral squamous cell carcinoma.**
Additional file 12:
**Script for the 3D MALDI imaging MS dataset of microbial colonies in a time course experiment.**

## Abbreviations

2D: Two-dimensional
3D: Three-dimensional
DESI: Desorption electro-spray ionization
DHB: Dihydroxybenzoic acid
GB: Gigabyte
H&E: Haematoxylin & eosin
*m/z*: Mass-to-charge ratio
MALDI: Matrix-assisted laser desorption/ ionization
MS: Mass spectrometry
OSCC: Oral squamous cell carcinoma
SA: Sinapinic acid
TB: Terabyte
TOF: Time of flight

## References

[1] Seeley EH, Caprioli RM (2012). 3D imaging by mass spectrometry: a new frontier. Anal Chem.

[2] Stoeckli M, Chaurand P, Hallahan DE, Caprioli RM (2001). Imaging mass spectrometry: a new technology for the analysis of protein expression in mammalian tissues. Nat Med.

[3] Watrous JD, Alexandrov T, Dorrestein PC (2011). The evolving field of imaging mass spectrometry and its impact on future biological research. J Mass Spectrom.

[4] Watrous JD, Dorrestein PC (2011). Imaging mass spectrometry in microbiology. Nat Rev Microbiol.

[5] Stoeckli M, Staab D, Staufenbiel M, Wiederhold K-H, Signor L (2002). Molecular imaging of amyloid beta peptides in mouse brain sections using mass spectrometry. Anal Biochem.

[6] Karas M, Hillenkamp F (1988). Laser desorption ionization of proteins with molecular masses exceeding 10,000 daltons. Anal Chem.

[7] Karas M, Bachmann D, el Bahr U, Hillenkamp F (1987). Matrix-assisted ultraviolet laser desorption of non-volatile compounds. Int J Mass Spectrom Ion Processes.

[8] Takats Z, Wiseman JM, Gologan B, Cooks RG (2004). Mass spectrometry sampling under ambient conditions with desorption electrospray ionization. Science.

[9] Alexandrov T (2012). MALDI imaging mass spectrometry: statistical data analysis and current computational challenges. BMC Bioinformatics.

[10] Jones EA, van Remoortere A, van Zeijl RJ, Hogendoorn PC, Bovée JV, Deelder AM (2011). Multiple statistical analysis techniques corroborate intratumor heterogeneity in imaging mass spectrometry datasets of myxofibrosarcoma. PLoS One.

[11] Alexandrov T, Becker M, Guntinas-Lichius O, Ernst G, von Eggeling F (2013). MALDI-imaging segmentation is a powerful tool for spatial functional proteomic analysis of human larynx carcinoma. J Cancer Res Clin Oncol.

[12] Alexandrov T, Kobarg JH (2011). Efficient spatial segmentation of large imaging mass spectrometry datasets with spatially aware clustering. Bioinformatics.

[13] Palmer AD, Bunch J, Styles IB (2013). Randomized approximation methods for the efficient compression and analysis of hyperspectral data. Anal Chem.

[14] Schramm T, Hester A, Klinkert I, Both J-P, Heeren R, Brunelle A (2012). imzML—a common data format for the flexible exchange and processing of mass spectrometry imaging data. J Proteomics.

[15] Oetjen J, Veselkov K, Watrous J, McKenzie JS, Becker M, Hauberg-Lotte L, et al. Supporting materials for “Benchmark datasets for 3D MALDI- and DESI-Imaging Mass Spectrometry.” GigaScience Database. 2015; http://dx.doi.org/10.5524/100131 10.1186/s13742-015-0059-4 PMC4418095 25941567

[16] The HDF group: http://www.hdfgroup.org/HDF5/ (1997) Accessed 27 Mar 2015.

[17] Oetjen J, Aichler M, Trede D, Strehlow J, Berger J, Heldmann S (2013). MRI-compatible pipeline for three-dimensional MALDI imaging mass spectrometry using PAXgene fixation. J Proteomics.

[18] Yang Y-L, Xu Y, Straight P, Dorrestein PC (2009). Translating metabolic exchange with imaging mass spectrometry. Nat Chem Biol.

[19] Watrous JD, Phelan VV, Hsu C-C, Moree WJ, Duggan BM, Alexandrov T (2013). Microbial metabolic exchange in 3D. ISME J.

[20] Gerbig S, Golf O, Balog J, Denes J, Baranyai Z, Zarand A (2012). Analysis of colorectal adenocarcinoma tissue by desorption electrospray ionization mass spectrometric imaging. Anal Bioanal Chem.

[21] MALDI MSI Interest Group: Msimaging. http://www.maldi-msi.org (2011). Accessed 27 Mar 2015.

[22] McKenzie JS: 3D Massomics Github repository. https://github.com/jsmckenzie/3DMassomics (2014) Accessed 27 Mar 2015.

[23] Veselkov KA, Mirnezami R, Strittmatter N, Goldin RD, Kinross J, Speller AV (2014). Chemo-informatic strategy for imaging mass spectrometry-based hyperspectral profiling of lipid signatures in colorectal cancer. Proc Natl Acad Sci U S A.

[24] Rohner TC, Staab D, Stoeckli M (2005). MALDI mass spectrometric imaging of biological tissue sections. Mech Ageing Dev.

[25] Klinkert I, Chughtai K, Ellis SR, Heeren R (2014). Methods for full resolution data exploration and visualization for large 2D and 3D mass spectrometry imaging datasets. Int Journal Mass Specrom.

[26] Robichaud G, Garrard KP, Barry JA, Muddiman DC (2013). MSiReader: an open-source interface to view and analyze high resolving power MS imaging files on Matlab platform. J Am Soc Mass Spectrom.

[27] Race AM, Stylers, IB, Bunch, J.: imzMLConverter. www.imzmlconverter.co.uk (2012) Accessed 27 Mar 2015.

[28] Race AM, Styles IB, Bunch J (2012). Inclusive sharing of mass spectrometry imaging data requires a converter for all. J Proteomics.

