# Supplementary material for: Benchmark datasets for 3D MALDI- and DESI-imaging mass spectrometry
Source: Gigascience. 2015 May 4;4:20. doi: 10.1186/s13742-015-0059-4 (PMC4418095; doi:10.1186/s13742-015-0059-4)
Supplement: Additional file 1: — Information about the 3D imaging MS datasets. [file 13742_2015_59_MOESM1_ESM.pdf]

Data information for the data note entitled 'Benchmark datasets for 3D MALDI- and DESI-Imaging Mass Spectrometry' submitted to the GigaScience journal

[1] Janina Oetjen , [2] Kirill Veselkov, [3] Jeramie Watrous, [2] James S. McKenzie, [4] Michael Becker, [5] Lena Hauberg-Lotte,[2] Nicole Strittmatter, [2] Anna K. Mróz, [7,8,9,10] Franziska Hoffmann, [5,6] Dennis Trede, [6] Jan Hendrik Kobarg, [11] Andrew Palmer, [6] Stefan Schiffler, [6] Klaus Steinhorst, [12] Michaela Aichler, [13] Robert Goldin, [8] Orlando Guntinas-Lichius, [7,8,9,10] Ferdinand von Eggeling, [5] Herbert Thiele, [14] Kathrin Mädler, [12] Axel Walch, [11] Peter Maass, [15] Pieter Dorrestein, [2] Zoltan Takats, [1,5,6,11,15,16] Theodore Alexandrov

[1] MALDI Imaging Lab, University of Bremen, Bremen, Germany; [2] Department of Surgery and Cancer, Faculty of Medicine, Imperial College London, London, United Kingdom; [3] Department of Medicine, Biomedical Research Facility II, University of California, San Diego, United States of America; [4] Bruker Daltonik GmbH, Bremen, Germany; [5] SCiLS Research, Bremen, Germany; [6] SCiLS GmbH, Bremen, Germany; [7] Institute of Physical Chemistry, Friedrich-Schiller-University Jena, Jena, Germany; [8] Department of Otorhinolaryngology, Jena University Hospital, Jena, Germany; [9] Leibnitz Institute of Photonic Technology (IPHT), Jena, Germany; [10] Jena Center for Soft Matter (JCSM), Friedrich-Schiller-University Jena, Jena, Germany; [11] Center for Industrial Mathematics, University of Bremen, Bremen, Germany; [12] Research Unit Analytical Pathology, Institute of Pathology, Helmholtz Center Munich, Munich, Germany; [13] Department of Medicine, Faculty of Medicine, Imperial College London; [14] Islet Research Lab, Center for Biomolecular Interactions, University of Bremen, Bremen, Germany; [15] Skaggs School of Pharmacy & Pharmaceutical Sciences, University of California, San Diego, USA; [16] European Molecular Biology Laboratory, Heidelberg, Germany.

Summary

Three-dimensional imaging mass spectrometry (3D imaging MS) is a novel technique for multiplexed molecular imaging. We are freely providing five different 3D imaging MS datasets from two mass spectrometric techniques to stimulate the development of data processing, interpretation and evaluation methods. The data is available in the GigaScience database. Four datasets were generated by data acquisition using matrix assisted laser desorption/ ionization imaging mass spectrometry (MALDI imaging MS) while the data for the fifth dataset was collected by desorption electrospray ionization imaging mass spectrometry (DESI imaging MS). With this document we want to provide summarized information on the data itself.

3D MALDI imaging MS datasets

All datasets which were collected by application of the MALDI imaging MS technique are available as files converted into the imzML format, an open and community-accepted format for exchange of imaging MS data [1]. For more information about the imzML format, its structure and instructions on how to read it, please go to http://www.maldi-msi.org/.

A summary on the information about the four MALDI imaging MS datasets is provided in Table 1.

Table 1. Overview of the 3D MALDI imaging MS datasets.

Dataset name	Format	Size [GB]	Organism	Strain	Sample type	Acknowledgement*
3DMouseKidney	imzML	41.1	*Mus musculus*	C57BL/6	Kidney	BMBF grant 01IB10004A-F
3D_Mouse_Pancreas	imzML	26.2	*Mus musculus*	C57BL/6	Pancreas	EU FP7 grant 305259
3D_OSCC	imzML	24.9	*Homo sapiens*		Oral squamous cell carcinoma	BMBF grant 01IB10004A-F
Microbe_Interaction _3D_Timecourse_LP (short name: 3D_Timecourse)	imzML	2.7	*Streptomyces coelicolor* and *Bacillus subtilis*	A3(2) PY79	Microbial colonies on agar plate	EU FP7 grant 305259

*Abbreviations: MALDI, matrix assisted laser desorption/ ionization; DESI, desorption electrospray ionization; BMBF, Bundesministerium für Bildung und Forschung, EU FP7, European Union 7th Framework Program.

More detailed information on the individual MALDI imaging datasets is provided in Table 2 which includes for each dataset information on the responsible authors, the sample as well as acquisition details and spectral information.

Table 2. Detailed information on the 3D MALDI imaging datasets

	3D_MouseKidney	3D_Mouse_Pancreas	3D_OSCC	3D_Timecourse
Authors	JO, MA, LHL, MB, AW	JO, KM, TA	JO, LHL, FH, FvE, OLG	JW, JO, PD
Number of sections	75	29	58	18
Preservation technique	PAXgene®	PAXgene®	Fresh frozen	Dried
MALDI MS mode	Linear, positive	Linear, positive	Linear, positive	Linear, positive
Mass range	2,000-20,000	1,600-15,000	2,000-20,000	0-4,000
Number of spectra	1,362,830	497,225	825,558	17,677
Intensity values per spectrum	7,680	13312	7,680	40,299
Voxel size	50×50×50	60×60×5	60×60×60	400×400×1500
Preprocessing	Yes	No	Yes	No

3D DESI imaging MS dataset

The dataset of the human colorectal adenocarcinoma was collected by application of the DESI imaging MS technique. We provide this dataset both in the imzML format (individual sections stored separately) and in its 3D processed form as an HDF5 file. HDF5 is a flexible and platform independent format for storing large datasets. More information about HDF5 along with example code for a range of programming languages can be found at http://www.hdfgroup.org/HDF5/. An overview about these data is provided in Table 3.

Table 3. Overview of the DESI imaging MS data.

Dataset name	Format	Type	Size [GB]
Colorectal_Adenocarcinoma	HDF5	3D imaging MS	1.1
ColAd_individual	imzML	2D imaging MS	0.6

The imzML data files were named with respect to the section number and location of the section on the glass slide. Since four sections were mounted on one glass slide, each file contains the DESI data of four sections. The tissue was sectioned at a thickness of 10 µm and data was acquired from every 10th section producing voxel of 100 µm^3. For example, in the file named '*120TopL, 90TopR, 110BottomL, 100BottomR-centroid.imzML*' the top-right section is the 90th section cut from the sample at a depth of 900 µm.

More detailed information on the 3D DESI imaging MS dataset is documented in Table 4. It provides a summary on the responsible authors for this dataset and lists information about the sample and on the acquisition details.

Table 4. Detailed information on the 3D DESI imaging dataset which is available as HDF5 file.

	Colorectal_Adenocarcinoma
Authors	KV, JMK, NS, AM, ZT
Number of sections	26
Preservation technique	Fresh frozen
DESI MS polarity	Negative
Mass range	200-1,050
Voxel size	100×100×100
Preprocessing	Yes

We would like to acknowledge the support the European Union 7th Framework Program grant 305259 for generation of the DESI imaging MS data.

Reference

1. Schramm T, Hester A, Klinkert I, Both J-P, Heeren R, Brunelle A, Laprévote O, Desbenoit N, Robbe M-F, Stoeckli M, others: **imzML—a common data format for the flexible exchange and processing of mass spectrometry imaging data**. *Journal of proteomics* 2012, **75**:5106–5110.
