# Supplementary material for: Benchmark datasets for 3D MALDI- and DESI-imaging mass spectrometry
Source: Gigascience. 2015 May 4;4:20. doi: 10.1186/s13742-015-0059-4 (PMC4418095; doi:10.1186/s13742-015-0059-4)
Supplement: Additional file 2: — Supplementary information with figures S1-S5. [file 13742_2015_59_MOESM2_ESM.pdf]

Supplementary information for data note article entitled

“Benchmark datasets for 3D MALDI- and DESI-Imaging Mass Spectrometry”

by Oetjen et al. (2015)
submitted to the Gigascience Journal

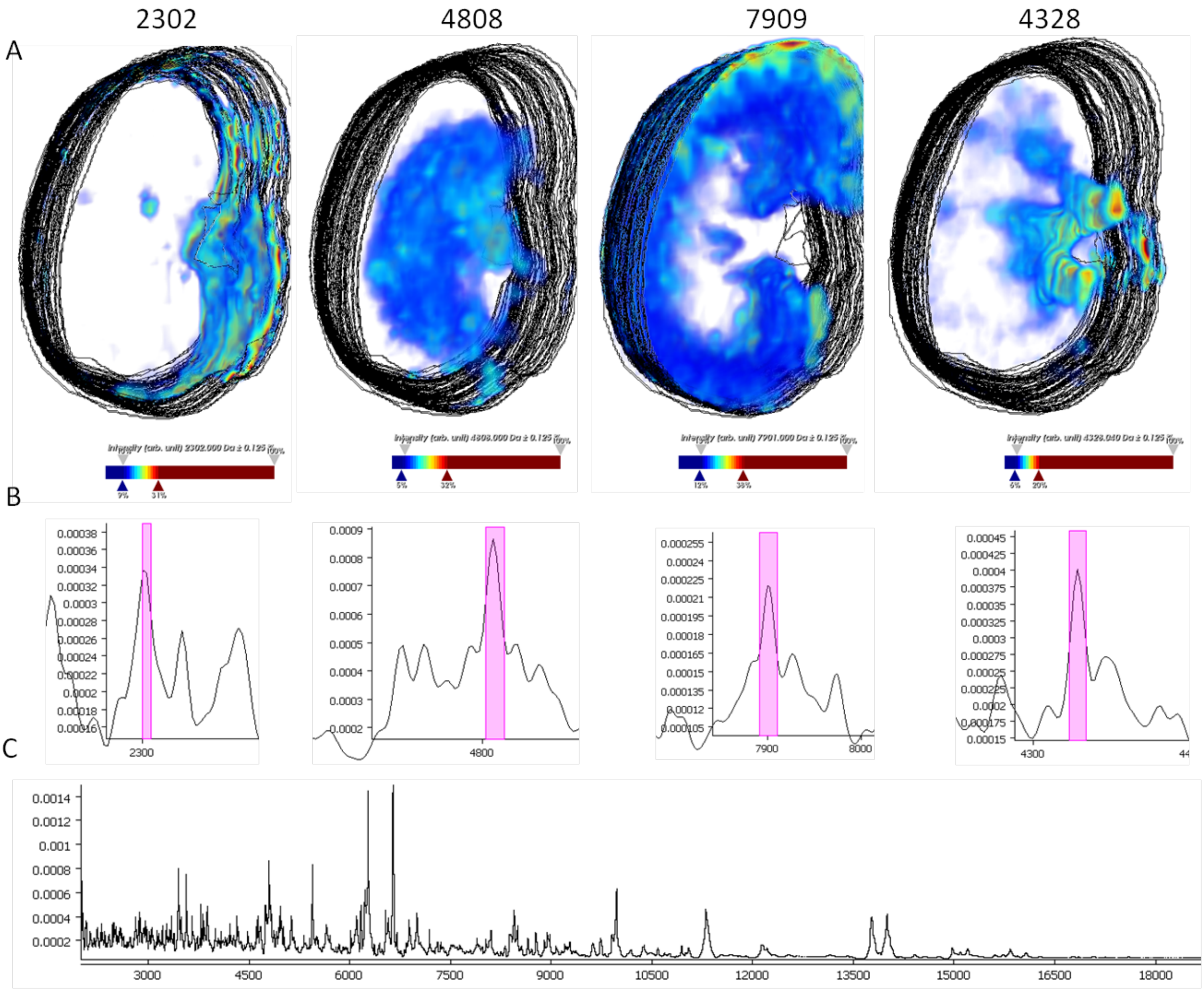

Supplementary Figure S1. Overview of the 3D mouse kidney dataset. (A) Visualization of m/z-values 2302, 4808, 7901 and 4328, respectively, in the software SCiLS Lab version 2014b after preprocessing. The intensity distributions were visualized in volume mode with strong denoising applied. The intensities are represented with a false-color map with the blue color corresponding to low intensity and the red color corresponding to high intensity; low-intensity voxels were made transparent, color bars represent the visible intensity range in arbitrary units. The section outline is represented by a black line. (B) Zoom into the mean spectrum at the m/z-values shown in (A). The mean spectrum for the entire dataset over the complete m/z-range is shown in (C).

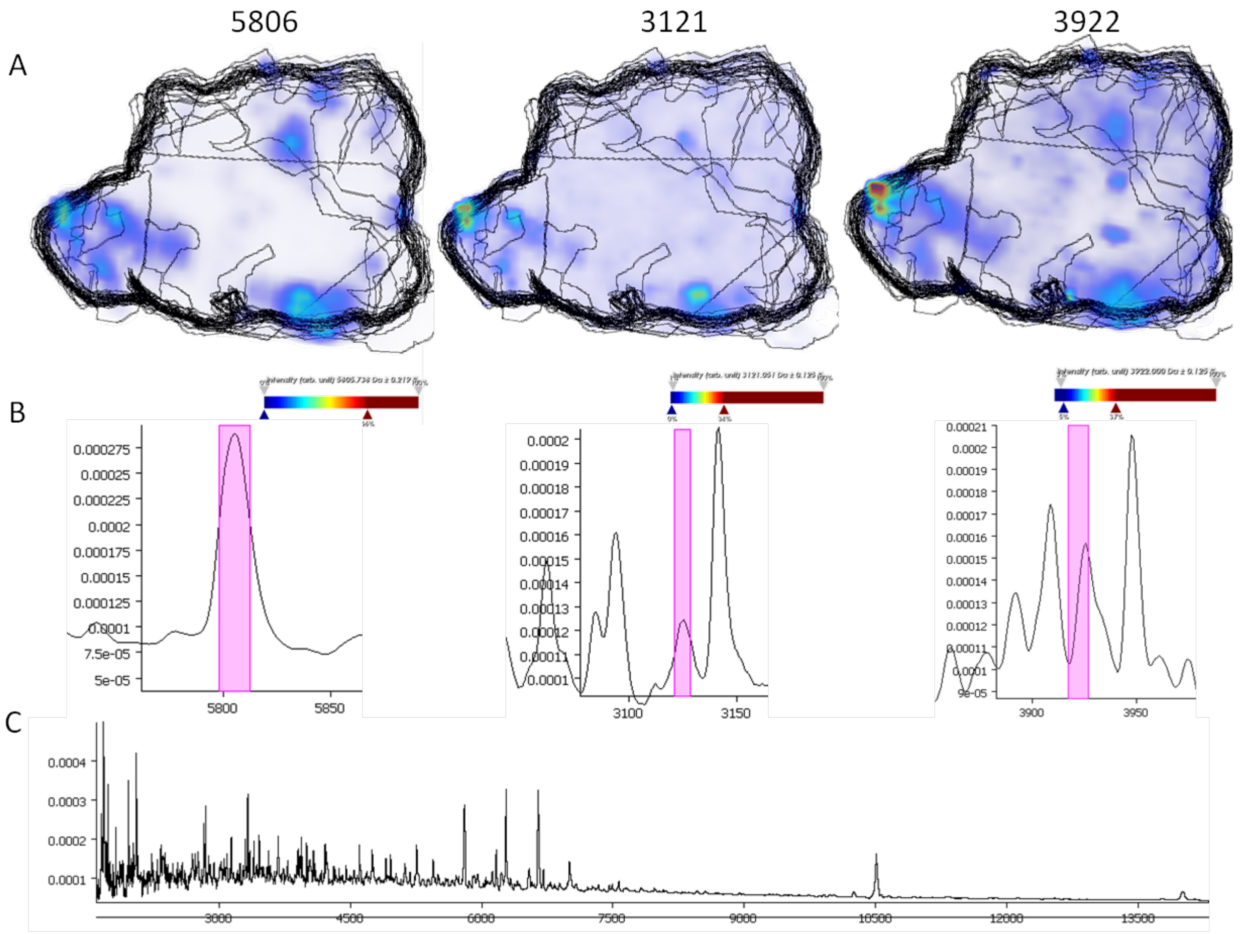

Supplementary Figure S2. Overview of the 3D mouse pancreas dataset. (A) Intensity distribution of insulin (m/z-value 5806), insulin c-peptide (m/z-value 3121) and islet amyloid polypeptide (m/z-value 3922) in the 3D pancreas model visualized with SCiLS Lab version 2014b in volume mode with strong denoising applied. The data was preprocessed using the SCiLS Lab preprocessing pipeline. The intensities are represented with a false-color map with the blue color corresponding to low intensity and the red color corresponding to high intensity; low-intensity voxels were made transparent, color bars represent the visible intensity range in arbitrary units. The section outline is represented by a black line. (B) Zoom into the mean spectrum at the m/z-values shown in (A). The mean spectrum for the entire dataset over the complete m/z-range is shown in (C).

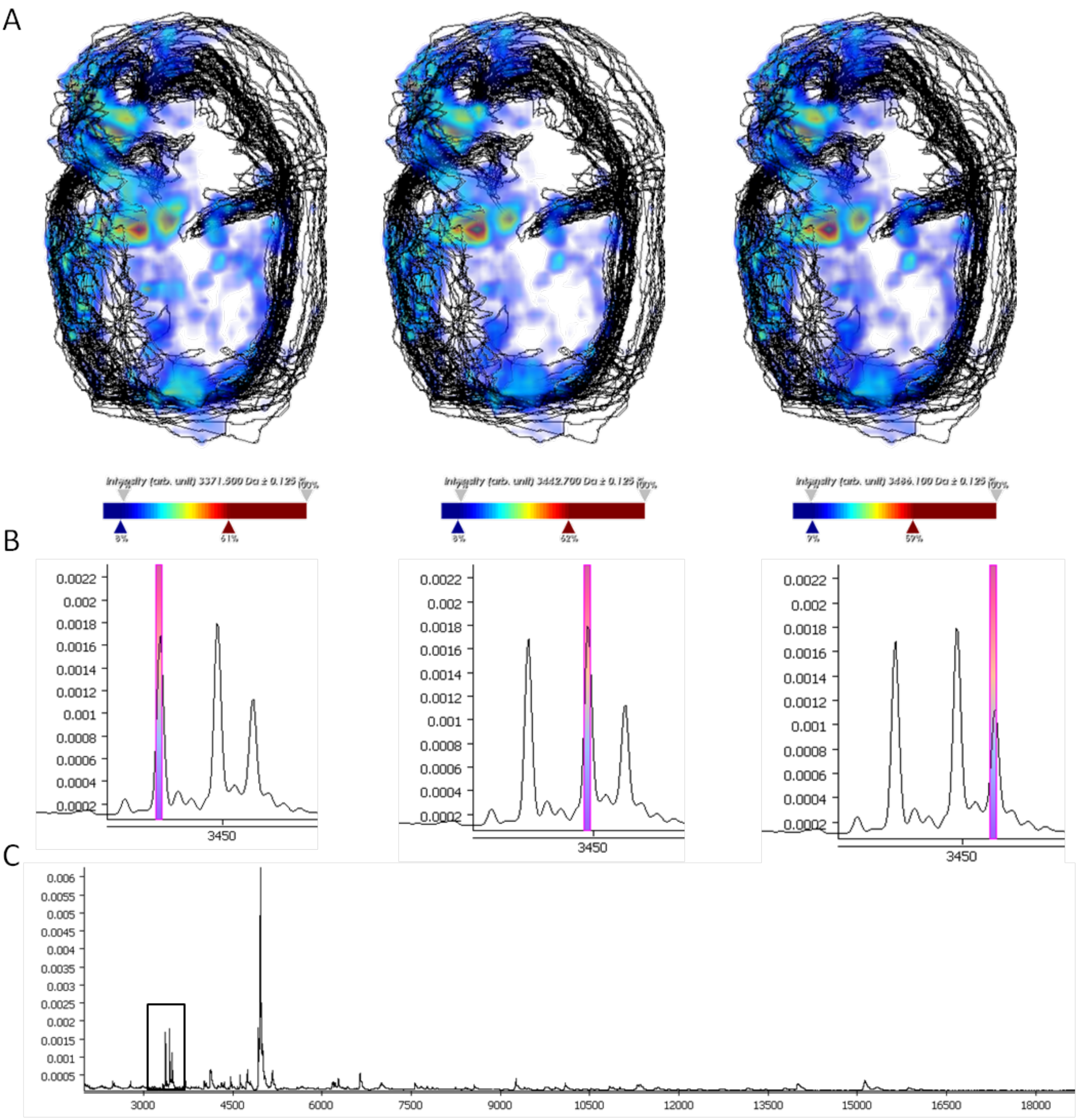

Supplementary Figure S3. Overview of the 3D human oral squamous cell carcinoma dataset. (A) Visualization of m/z-values 3372, 3443, 3486, respectively, which represent the putative defensins HNP1-3. The data was preprocessed using the software SCiLS Lab version 2014b. The intensity distributions were visualized in volume mode with strong denoising applied. The intensity is represented with a false-color map with the blue color corresponding to low intensity and the red color corresponding to high intensity; low-intensity voxels were made transparent, color bars represent the visible intensity range in arbitrary units. The section outline is represented by a black line. (B) Zoom into the mean spectrum at the m/z-values shown in (A). The mean spectrum for the entire dataset over the complete m/z-range is shown in (C). The box indicates the m/z-range enlarged in (B).

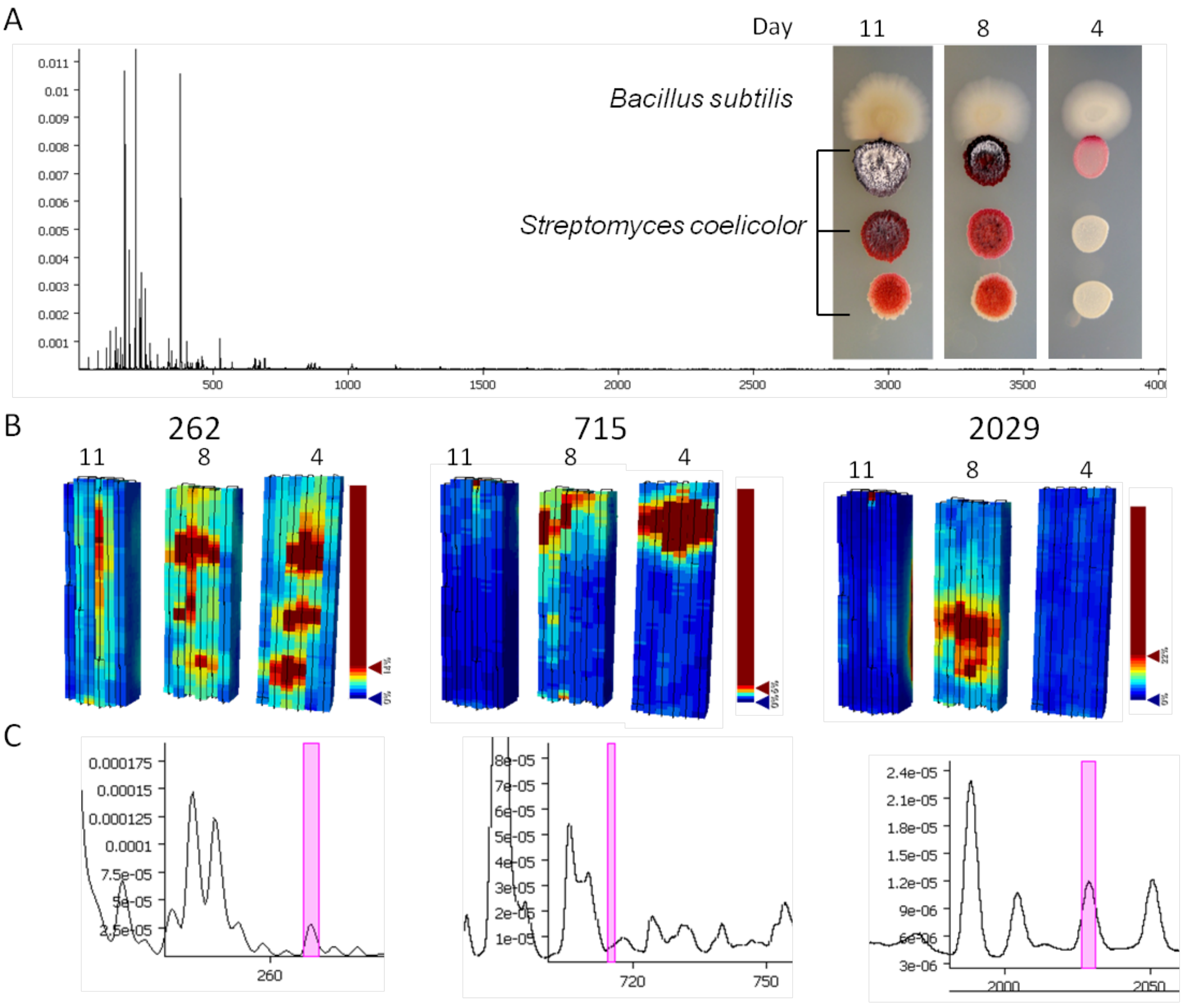

Supplementary Figure S4. Overview of the 3D dataset of microbial colonies from a time course experiment (A). Mean spectrum of the entire dataset over the complete m/z-range and fotograph of the microbial colonies from *Bacillus subtilis* and *Streptomyces coelicolor* after 4, 8 and 11 days after co-inoculation. Visualization of the 3D data using the software SCiLS Lab version 2014b after preprocessing (B). The intensity distributions of three m/z-values (262, 715 and 2029 as indicated) are shown after image denoising in slice view mode. The intensity is represented with a false-color map with the blue color corresponding to low intensity and the red color corresponding to high intensity; color bars represent the visible intensity range in arbitrary units. The section outline is represented by a black line and the time after co-inoculation in days is given above the individual 3D models. Zoom into the mean spectrum (C) at the m/z-values shown in (B).

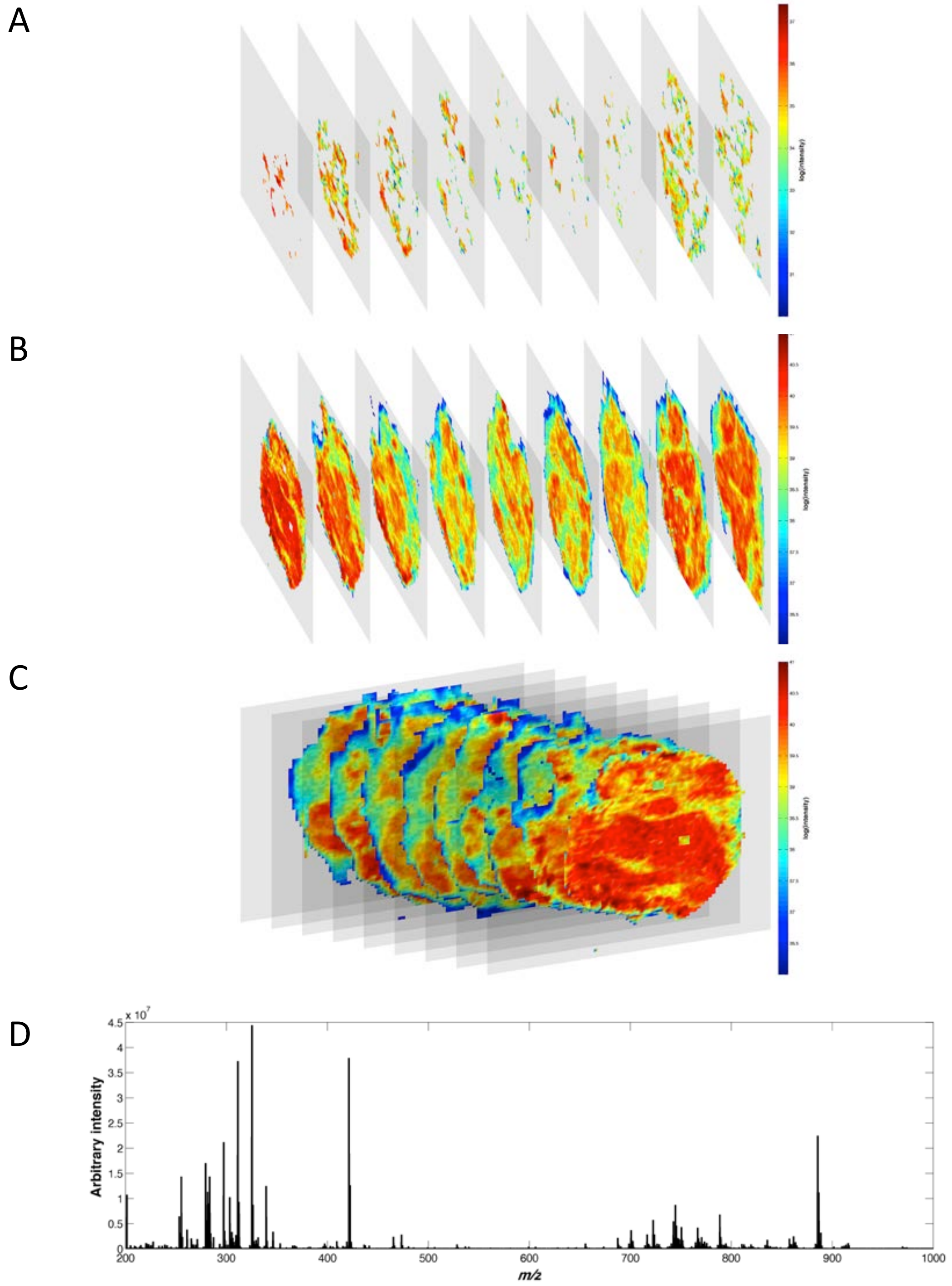

Supplementary Figure S5. An overview of the DESI-acquired colorectal adenocarcinoma. In (A) and (B) ion distributions across nine tissue sections are shown for ions of *m/z* value 899.58 and 885.55, respectively. Image (C) is a rotated view of (B). The intensities are denoted by the adjacent colour bar, and thresholding has been applied according to the range specified. The images were prepared using Matlab 2014b, using the functions provided in the 3DMassomics Github repository (for details see the main text). An average mass spectrum across all pixels is given in (D).
